# High-Fat Diet-Induced Obesity Enhances Small Intestinal Glucose and NaCl Absorption Through Selective Transporter Reprogramming

**DOI:** 10.3390/ijms27093961

**Published:** 2026-04-29

**Authors:** Balasubramanian Palaniappan, Niraj Nepal, John Crutchley, Subha Arthur

**Affiliations:** 1 Department of Biomedical Sciences, Joan C Edwards School of Medicine, Marshall University, Huntington, WV 25701, USA; nnepal@osteo.wvsom (N.N.); arthursu@marshall.edu (S.A.); 2 Biomedical Sciences, West Virginia School of Osteopathic Medicine, Lewisburg, WV 24901, USA; 3 Marshall Institute for Interdisciplinary Research, Marshall University, Huntington, WV 25703, USA; crutchleyj@marshall.edu

**Keywords:** metabolic dysfunction, obesity, diet-induced obesity, SGLT1, DRA, PAT1, NHE3

## Abstract

Metabolic dysfunction, a hallmark of diet-induced obesity (DIO), is increasingly attributed to alterations in intestinal nutrient and electrolyte transport. Yet the mechanisms that drive obesity-associated functional alterations of intestinal transporters remain incompletely understood. In this context, the effects of a high-fat diet (HFD) induced obesity on sodium-dependent glucose co-transporter 1 (SGLT1), Na^+^/H^+^ exchanger 3 (NHE3), and Cl^−^/HCO_3_^−^ exchangers (DRA/PAT1), the primary glucose, sodium, and chloride absorptive pathways in mice small intestinal villus cells, were investigated. SGLT1 activity significantly increased in intact villus cells and brush border membrane vesicles (BBMV) from HFD-fed mice. Kinetic analysis demonstrated reduced Km without a change in Vmax, indicating enhanced transporter affinity. Notably, SGLT1 mRNA and protein expression, including BBM localization, were unchanged. Basolateral Na^+^/K^+^-ATPase activity was decreased, excluding enhanced Na^+^ gradient generation as the mechanism for SGLT1 stimulation. In contrast, DRA/PAT1 activity was significantly increased in HFD-fed mice, and kinetic studies revealed elevated Vmax without a change in Km, indicating increased transport capacity. DRA/PAT1 mRNA, total protein, and BBM expression were all significantly elevated. NHE3 activity and expression remained unchanged. These findings demonstrate that DIO enhances intestinal glucose absorption by increasing SGLT1 affinity and chloride absorption by upregulating DRA/PAT1 transcription. These transporter-specific alterations may amplify nutrient absorption and contribute to metabolic dysregulation in obesity.

## 1. Introduction

Obesity has emerged as one of the most significant global health challenges, with prevalence increasing steadily over the past several decades. Excess adiposity is strongly associated with the development of metabolic disorders including insulin resistance, type 2 diabetes mellitus, dyslipidemia, and cardiovascular disease [1,2,3,4,5,6,7,8]. While the systemic metabolic consequences of obesity have been extensively studied in tissues such as liver, skeletal muscle, and adipose tissue, the contribution of the gastrointestinal tract to metabolic dysregulation in obesity is increasingly recognized [9,10,11,12,13,14]. The intestine is now understood to play an active role in metabolic regulation through altered nutrient absorption, hormone secretion, immune signaling, altered bile acid composition and interactions with the microbiome and its metabolites [11,15,16,17,18]. In obesity, alterations in nutrient absorptive processes are known to induce nutrient-induced intestinal adaptation in obesity thus driving the differences in nutrient processing in lean versus obese individuals [19]. This intestinal adaptation may therefore significantly influence systemic nutrient and electrolyte homeostasis in obesity.

The small intestine is the principal site of nutrient absorption and serves as the first physiological barrier regulating entry of dietary substrates into the systemic circulation [20,21]. Enterocytes located along the intestinal villi express specialized transport proteins responsible for the uptake of carbohydrates, amino acids, electrolytes, and water [22]. Among these transporters, sodium-glucose cotransporter-1 (SGLT1; *SLC5A1*) plays a central role in intestinal glucose absorption. SGLT1 mediates the electrogenic co-transport of glucose together with sodium ions across the brush border membrane (BBM) of enterocytes, allowing glucose to be absorbed against its concentration gradient [23,24]. This process is essential for efficient dietary carbohydrate assimilation and contributes significantly to postprandial glycemic excursions. The activity of SGLT1 depends critically on the inward sodium gradient generated by the basolateral Na^+^/K^+^-ATPase pump. This enzyme actively extrudes three sodium ions from the cell in exchange for two potassium ions, thereby maintaining the electrochemical gradient required for sodium-coupled nutrient transport. Disruption of this gradient can directly affect the function of Na^+^-dependent transporters, including SGLT1 and other nutrient transport systems. Regulation of Na^+^/K^+^-ATPase activity, therefore, represents an important determinant of intestinal absorptive capacity [23].

In addition to nutrient absorption, the small intestine plays a major role in maintaining electrolyte and fluid balance. Electroneutral NaCl absorption in the intestinal epithelium is primarily mediated by the coordinated activity of the Na^+^/H^+^ exchanger 3 (NHE3; *SLC9A3*) and apical Cl^−^/HCO_3_^−^ exchangers. NHE3 facilitates sodium absorption by exchanging intracellular protons for luminal sodium ions, while chloride uptake occurs through members of the *SLC26* family of anion exchangers. Two major apical chloride exchangers involved in this process are downregulated in adenoma (DRA; *SLC26A3*) and putative anion transporter-1 (PAT1; *SLC26A6*). These transporters operate in concert to mediate electroneutral NaCl absorption and thereby regulate intestinal fluid transport and electrolyte homeostasis [25]. DRA has emerged as a particularly important regulator of intestinal chloride absorption. Mutations in the DRA gene cause congenital chloride diarrhea, underscoring its essential role in electrolyte transport [26,27,28]. Beyond hereditary disease, alterations in DRA activity have also been reported in inflammatory bowel disease, infectious diarrhea, and other gastrointestinal disorders [29,30,31,32].

Intestinal transporters are highly responsive to dietary composition. Adaptive regulation allows the intestine to adjust its absorptive capacity in response to variations in nutrient intake. For example, high-carbohydrate diets have been shown to increase SGLT1 expression and glucose absorption capacity, while fasting or low carbohydrate intake can suppress transporter expression [33,34,35,36]. Similarly, electrolyte transporters such as NHE3 and DRA can be regulated by hormonal, neural, and inflammatory signals [29,37,38,39,40]. These adaptive responses allow the intestine to maintain efficient nutrient and electrolyte absorption under changing physiological conditions.

Diet-induced obesity (DIO), commonly produced in experimental models through chronic consumption of a high-fat diet (HFD), reproduces many features of human metabolic syndrome including hyperglycemia, insulin resistance, and systemic inflammation [41,42]. Recent studies have suggested that obesity may also induce structural and functional adaptations within the intestinal epithelium. These changes include increased intestinal permeability, altered microbiome composition, and modifications in nutrient transporter expression [43,44,45]. However, the effects of obesity on intestinal transporter kinetics and membrane localization remain incompletely understood. It remains unclear how HFD-induced obesity affects the coordinated function of glucose transporters and electrolyte exchangers in the small intestinal villus epithelium.

Previous studies have suggested that intestinal glucose absorption may be enhanced in obesity and diabetes, potentially contributing to exaggerated postprandial glycemia [46]. However, the mechanisms responsible for such changes, whether due to increased transporter expression, altered kinetic properties, or modifications in membrane trafficking, are still not fully defined, especially in DIO models of obesity. Similarly, although the regulation of NHE3 and DRA has been extensively studied in inflammatory and infectious conditions, less is known about how metabolic disorders induced by DIO influence their activity in the small intestine. Understanding these mechanisms is important because alterations in electrolyte transport may influence fluid balance, nutrient absorption efficiency, and overall intestinal physiology during metabolic disease. Given the central role of intestinal transporters in nutrient and electrolyte absorption, elucidating their regulation in DIO may provide important insights into mechanisms linking intestinal physiology with systemic metabolic homeostasis. Specifically, alterations in intestinal glucose transport could exacerbate hyperglycemia, while changes in NaCl absorption pathways may affect epithelial transport capacity, intestinal fluid dynamics and potentially induce hypertension.

Therefore, the present study was designed to investigate how HFD alters the function and regulation of key intestinal transporters in villus cells of the small intestine. We specifically examined the activity and kinetic characteristics of SGLT1-mediated Na^+^-dependent glucose transport, as well as the activity of NHE3-mediated Na^+^/H^+^ exchange and Cl^−^/HCO_3_^−^ exchange mediated by DRA/PAT1. In addition, we assessed transporter expression at both the mRNA and protein levels and evaluated their localization within the brush border membrane. By integrating functional transport measurements with kinetic analysis and molecular expression studies, this work aims to define the mechanisms by which HFD-induced obesity modulates intestinal nutrient and electrolyte transport. Understanding these adaptations may provide new insights into the role of the small intestine in metabolic disease and may identify intestinal transport processes as potential therapeutic targets for obesity-associated metabolic disorders.

## 2. Results

### 2.1. SGLT1 Activity in Villus Cells of DIO Mice

**Figure 1:**
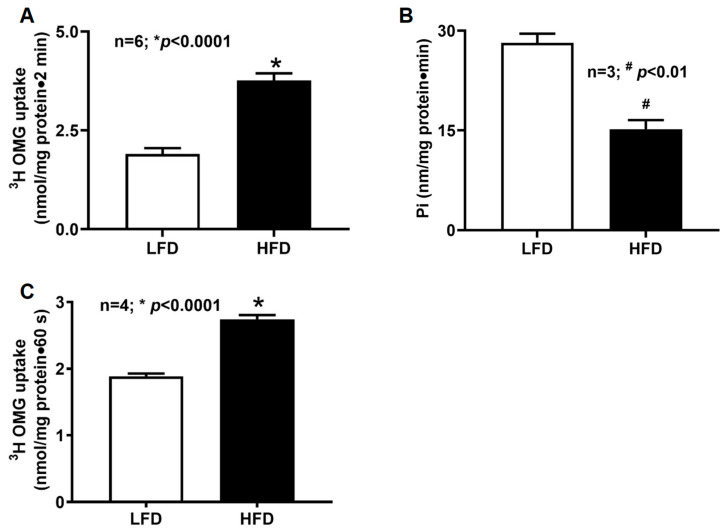
SGLT1 uptake and Na^+^/K^+^-ATPase activity in villus cells of DIO mice. (**A**,**C**) Phlorizin-sensitive, Na^+^-dependent ^3^H-OMG uptake was detected in intact and BBMV villus cells from both LFD- and HFD-fed mice. (**A**) Intact villus cells from HFD-fed mice exhibited a significant increase in SGLT1-mediated uptake compared with LFD controls; (**B**) Na^+^/K^+^-ATPase activity was quantified in villus-cell homogenates using an inorganic phosphate release assay. HFD-fed mice showed a significant reduction in Na^+^/K^+^-ATPase activity compared with LFD-fed mice; (**C**) BBMV from HFD-fed mice demonstrated significantly enhanced SGLT1-mediated uptake compared with LFD controls. LFD: low-fat diet; HFD: high-fat diet.

SGLT1-mediated glucose transport, measured as phlorizin-sensitive, Na^+^-dependent uptake of tritiated 3-O-methyl-D-glucose (^3^H-OMG), was markedly elevated in intact villus cells isolated from HFD-fed mice compared with LFD controls (Figure 1A; LFD: 1.9 ± 0.2 nmol/mg protein·2 min; HFD: 3.8 ± 0.2; *n* = 6, *p* < 0.01). This finding indicates that HFD feeding enhances intestinal glucose absorption, accompanied by increased Na^+^ absorption enabled by the Na^+^-coupled transport mechanism of SGLT1 in the villus cells.

### 2.2. Na^+^/K^+^-ATPase Activity in Villus Cells of DIO Mice

To determine whether increased SGLT1 activity was driven by changes in the basolateral Na^+^ gradient, Na^+^/K^+^-ATPase activity was measured in villus-cell homogenates. Surprisingly, Na^+^/K^+^-ATPase activity was significantly reduced in HFD-fed mice (HFD: 15.2 ± 1.4 nmol Pi/mg protein·min; LFD: 28.2 ± 1.4; *n* = 3, *p* < 0.01) (Figure 1B). These results demonstrate that the enhanced Na^+^-glucose co-transport observed in DIO is not attributable to an increased transcellular Na^+^ gradient.

### 2.3. BBM SGLT1 Activity in Villus Cells of DIO Mice

To determine whether DIO directly affects SGLT1 at the BBM level, SGLT1 activity was measured in villus-cell BBMV. SGLT1-mediated uptake was significantly higher in BBMV from HFD-fed mice compared with LFD controls (Figure 1C; HFD: 2.7 ± 0.07 nmol/mg protein·2 min; LFD: 1.9 ± 0.04; *n* = 4, *p* < 0.01). These findings indicate that obesity enhances glucose absorption by increasing SGLT1 activity at the BBM.

### 2.4. Kinetic Properties of BBM SGLT1 in DIO Mice

**Figure 2:**
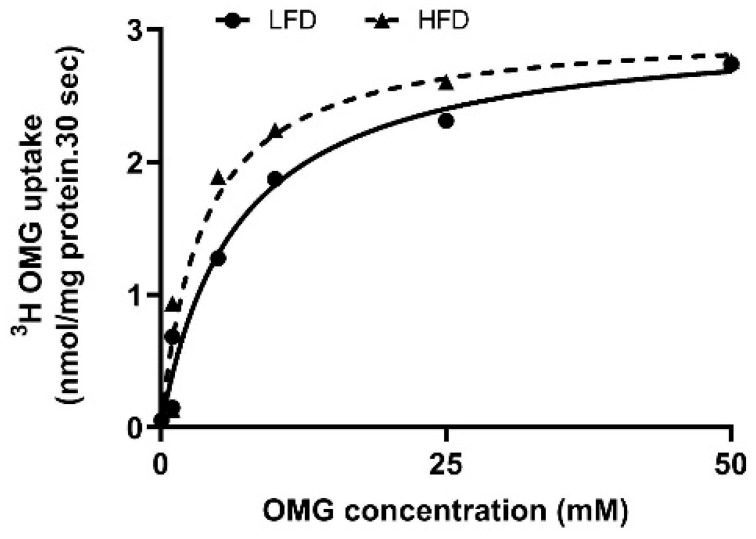
Kinetic analysis of SGLT1 activity in villus-cell BBMV from DIO mice. Increasing extravesicular OMG concentrations stimulated Na^+^-dependent ^3^H-OMG uptake, which reached saturation in both diet groups. Kinetic analysis revealed a significant increase in substrate affinity (1/K*_m_*) in BBMV from HFD-fed mice, whereas maximal transport velocity (V*_max_*) remained unchanged (Table 1).

Kinetic analyses were performed to define the functional mechanism underlying SGLT1 stimulation. Uptake was measured at 6 s, within the linear phase of Na^+^-dependent glucose transport. Increasing extravesicular OMG concentrations produced saturable uptake in both groups (Figure 2). Kinetic parameters (Table 1) revealed a significant increase in substrate affinity (lower K*_m_*) in HFD-fed mice, whereas V*_max_* remained unchanged. These findings indicate that DIO enhances SGLT1 activity primarily through increased transporter affinity rather than maximal transport capacity.

### 2.5. BBM NHE3 Activity in Villus Cells of DIO Mice

**Figure 3:**
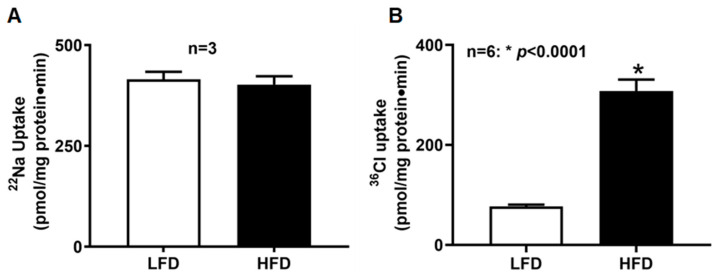
BBMV Na^+^/H^+^ and Cl^−^/HCO_3_^−^ exchange activities in villus cells of DIO mice. (**A**) Amiloride-sensitive, H^+^-gradient-dependent ^22^Na^+^ uptake, revealing NHE3 activity, was unchanged between LFD- and HFD-fed mice; (**B**) DIDS-sensitive, HCO_3_**^−^**-dependent ^36^Cl^−^ uptake was significantly increased in BBMV from HFD-fed mice compared with LFD controls. LFD: low-fat diet; HFD: high-fat diet.

To determine whether DIO affects Na^+^/H^+^ exchange, amiloride-sensitive ^22^Na^+^ uptake was measured in BBMV. NHE3 activity did not differ between LFD and HFD groups (Figure 3A; LFD: 414.9 ± 18.9 pmol/mg protein·2 min; HFD: 401.9 ± 20.9; *n* = 3). These results indicate that DIO does not alter BBM Na^+^/H^+^ exchange in villus cells.

### 2.6. BBM DRA/PAT1 Activity in Villus Cells of DIO Mice

Cl^−^/HCO_3_^−^ exchange activity was detected in both diet groups; however, villus cells from HFD-fed mice exhibited a marked increase in DRA/PAT1-mediated exchange (HFD: 308 ± 22.6 pmol/mg protein·min; LFD: 77.0 ± 3.5; *n* = 6, *p* < 0.0001) (Figure 3B). These findings demonstrate that DIO significantly stimulates Cl^−^/HCO_3_^−^ exchange in the small intestine.

### 2.7. Kinetic Properties of BBM DRA/PAT1 in DIO Mice

**Figure 4:**
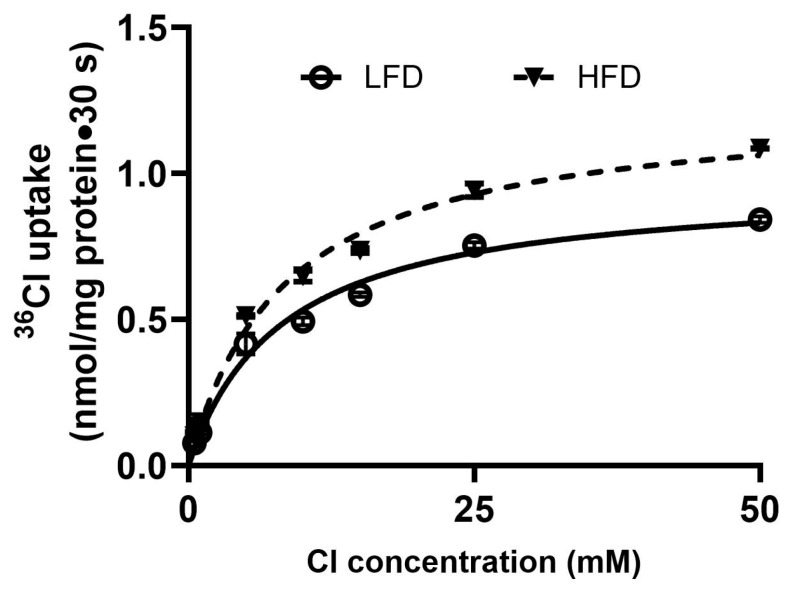
Kinetic studies of Cl^−^/HCO_3_^−^ exchange stimulation in DIO mice villus cell BBMV. In villus cell BBMV, as the extravesicular ^36^Cl^−^ concentration was increased, DIDS-sensitive, HCO_3_^−^-dependent ^36^Cl^−^ uptake was increased initially and subsequently saturated at all other conditions. The kinetic parameters derived from this study revealed that the affinity (1/K*_m_*) for ^36^Cl^−^ uptake was unchanged in HFD-fed mice villus BBMV. However, the maximal velocity (V*_max_*) rate was significantly increased (Table 2).

Kinetic studies were performed to determine the mechanism underlying enhanced DRA/PAT1 activities. Extracellular Cl^−^-dependent uptake increased in a concentration-dependent manner in both groups (Figure 4). K*_m_* values did not differ between HFD and LFD mice (Table 2), indicating unchanged affinity. In contrast, V*_max_* was significantly higher in HFD-fed mice compared with LFD controls (Table 2). Thus, DIO enhances DRA activity primarily by increasing maximal transport capacity.

### 2.8. mRNA Expression of SGLT1, NHE3, DRA, and PAT1 in Villus Cells of DIO Mice

**Figure 5:**
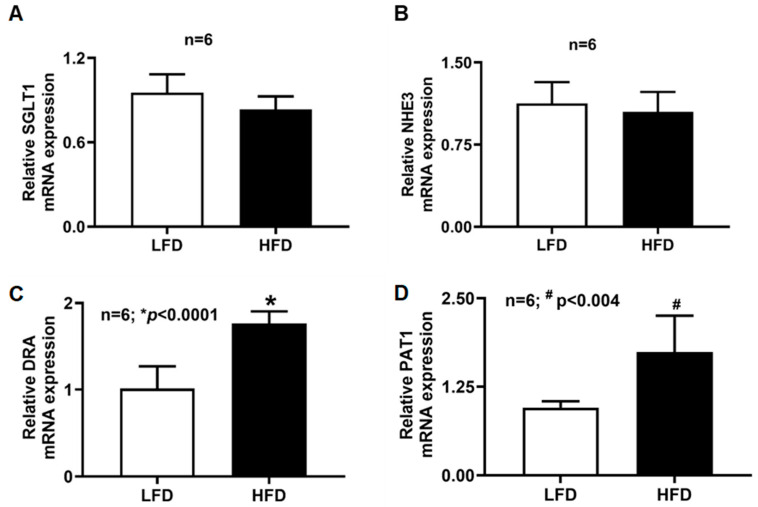
SGLT1 and DRA/PAT1 mRNA levels in the villus cells of DIO mice. (**A**) SGLT1 mRNA levels were unchanged in the villus cells between the two diet groups. (**B**) Like SGLT1, NHE3 mRNA levels were also unchanged. However, DRA and PAT1 mRNA levels (**C**,**D**) in the HFD-fed mice villus cells were significantly elevated compared to LFD-fed mice. LFD: low-fat diet; HFD: high-fat diet.

RT-qPCR analysis was performed to determine whether changes in transporter activity corresponded to altered gene expression (Figure 5). SGLT1 and NHE3 mRNA levels were unchanged between diet groups. In contrast, DRA and PAT1 mRNA levels were significantly increased in villus cells from HFD-fed mice. These results suggest transcriptional upregulation of DRA/PAT1 in DIO, whereas SGLT1 stimulation occurs through post-transcriptional or post-translational mechanisms.

### 2.9. SGLT1, DRA, and PAT1 Protein Expression Levels in the Villus Cell Lysate of DIO Mice

**Figure 6:**
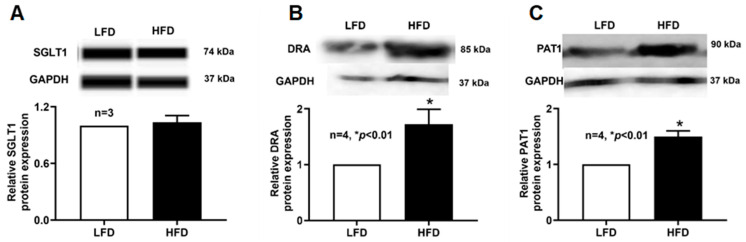
SGLT1 and DRA/PAT1 protein expressions in the villus cell lysate of DIO mice. Western blot studies revealed that the total cellular (**A**) SGLT1 protein expression of villus cell was unchanged between the diet groups. However, (**B**) DRA and (**C**) PAT1 protein expression of the villus cells were significantly increased in HFD-fed mice compared to LFD-fed mice. LFD: low-fat diet; HFD: high-fat diet.

Because mRNA abundance does not always correspond to functional protein levels, we next examined SGLT1, DRA, and PAT1 protein expression in whole-cell lysates. As shown in Figure 6, Western blot analysis revealed that SGLT1 protein levels were unchanged in villus cells from HFD-fed mice compared with LFD controls, consistent with the kinetic and mRNA data. In contrast, DRA and PAT1 protein levels were significantly elevated in villus cell lysates from HFD-fed mice, confirming the observed increases in DRA/PAT1 mRNA translate into higher total cellular protein abundance in DIO.

### 2.10. SGLT1, DRA, and PAT1 Protein Expression Levels in Villus Cell BBM of DIO Mice

**Figure 7:**
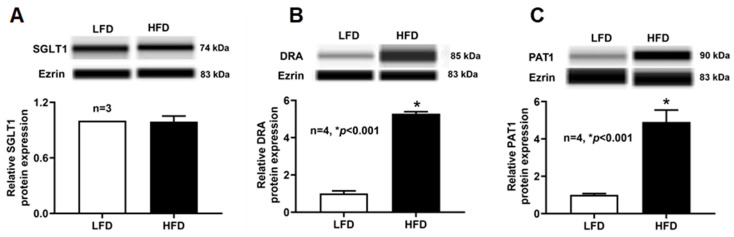
BBM SGLT1 and DRA/PAT1 protein expressions in the villus cells of DIO mice. Western blot studies revealed that the BBM (**A**) SGLT1 protein expression of villus cells was unchanged between the diet groups. However, (**B**) BBM DRA and (**C**) PAT1 protein expression were significantly increased in HFD-fed mice compared to LFD-fed mice villus cells. LFD: low-fat diet; HFD: high-fat diet.

We next assessed whether DIO alters the abundance of SGLT1, DRA, and PAT1 at the BBM, the functional site of these transporters. As shown in Figure 7, BBM SGLT1 protein levels did not differ between LFD- and HFD-fed mice, consistent with the unchanged V_max_ observed in kinetic studies. In contrast, BBM-localized DRA and PAT1 protein levels were markedly increased in villus cells from HFD-fed mice, indicating that the HFD feeding-associated upregulation of DRA/PAT1 at the cellular level is accompanied by enhanced localization of these transporters at the BBM, where they mediate ion exchange.

### 2.11. Plasma Sodium Concentration in DIO Mice

**Figure 8:**
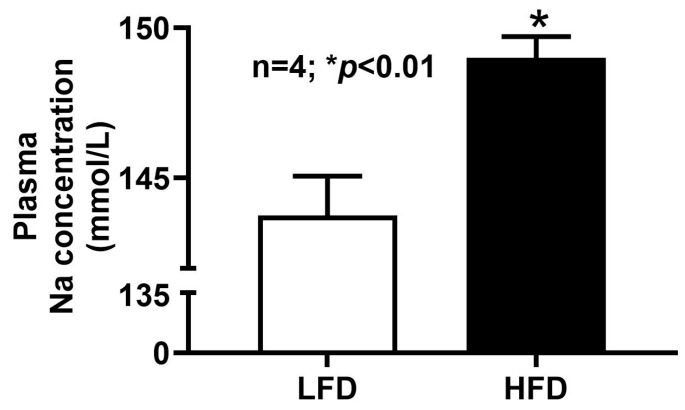
Plasma Na^+^ levels in DIO mice. Plasma Na^+^ levels were significantly increased in HFD mice compared to LFD mice. LFD: low-fat diet; HFD: high-fat diet.

Plasma sodium levels were measured in both HFD- and LFD-fed mice. Figure 8 shows that HFD-fed mice exhibited a significant elevation in circulating Na^+^ compared with LFD controls. Given that the absorption of glucose by the SGLT1 transporter is Na^+^-dependent, this increase in plasma Na^+^ is likely the consequence of the enhanced sodium-glucose absorption by SGLT1 in the villus cells of HFD-fed mice.

### 2.12. Plasma Glucose Concentration in DIO Mice

**Figure 9:**
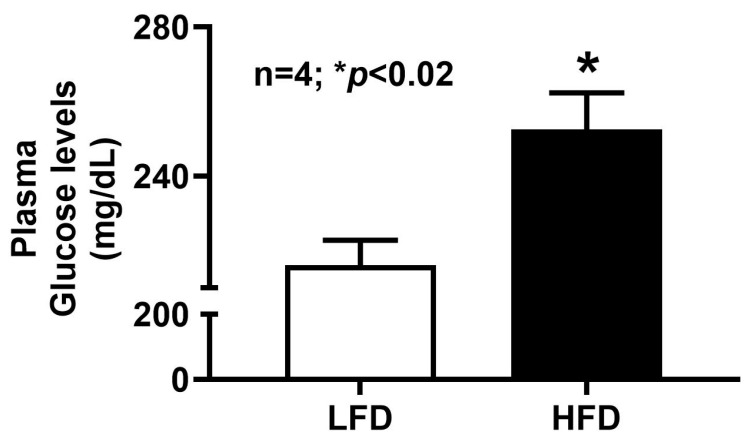
Blood glucose levels in DIO mice. HFD mice’s blood glucose concentrations were significantly elevated compared to LFD mice. LFD: low-fat diet; HFD: high-fat diet.

Plasma glucose levels were measured in both HFD- and LFD-fed mice to assess the impact of HFD feeding on systemic glucose homeostasis. As shown in Figure 9, HFD-fed mice exhibited substantially higher blood glucose concentrations compared with LFD controls. This increase parallels the elevated SGLT1 activity observed in villus cells from HFD-fed mice, suggesting that enhanced intestinal Na^+^-coupled glucose absorption contributes to the hyperglycemia associated with obesity.

## 3. Discussion

The present study demonstrates that DIO results in selective and transporter-specific alterations of small intestinal villus cell transport function through mechanistically distinct pathways. Rather than inducing a uniform enhancement of absorptive transport processes, DIO differentially regulates individual BBM transporters involved in nutrient and electrolyte absorption. Specifically, our findings show that HFD feeding enhances SGLT1-mediated glucose transport primarily through increased substrate affinity without altering transporter expression, while simultaneously augmenting DRA/PAT1-mediated chloride exchange via transcriptional upregulation and increased membrane abundance. In contrast, NHE3 activity and expression remain unchanged. These differential responses highlight the complexity of intestinal transport regulation in obesity and suggest that enterocyte transport machinery at the BBM undergoes selective adaptive changes rather than generalized activation in obesity.

SGLT1-mediated glucose uptake was significantly increased in intact villus cells and BBMV isolated from HFD mice. Importantly, kinetic analysis revealed a significant reduction in the apparent K_m_ with no change in maximal transport velocity V_max_. This kinetic profile indicates that the increased glucose transport results from enhanced substrate affinity rather than increased transporter abundance. Consistent with this interpretation, SGLT1 mRNA expression, total cellular protein levels, and BBM-localized protein abundance was unchanged between control and DIO groups. A reduction in K_m_ without changes in transporter abundance strongly suggests post-translational regulation of SGLT1 activity.

The lack of spatial resolution inherent to BBMV preparations, which pools membranes from heterogeneous enterocyte populations, limits the ability to determine whether the SGLT1 kinetic changes arise uniformly across the villus epithelium or reflect alterations in specific sub-domains of the BBM. Nevertheless, this observed reduction in K_m_ indicates qualitative modification of transporter behavior, suggesting that DIO optimizes glucose absorptive efficiency per transporter molecule. Such a mechanism may allow the intestine to increase glucose uptake without requiring increased protein synthesis or membrane trafficking.

Functionally, enhanced transporter affinity may promote more efficient glucose absorption at lower luminal glucose concentrations (as in HFD), potentially amplifying postprandial glucose uptake. Since SGLT1 functions as a sodium-dependent co-transporter, any upregulation of its activity enhances not only glucose uptake but also the coupled absorption of sodium. Interestingly, elevated systemic sodium levels were observed in the current study. However, the relationship between increased systemic electrolyte and glucose concentrations with alterations in intestinal transport function was not directly evaluated in this study. The broader physiological consequences of these findings remain to be defined in DIO.

In contrast to SGLT1, DRA/PAT1-mediated Cl^−^/HCO_3_^−^ exchange was increased through a fundamentally different mechanism. Kinetic analysis revealed a significant increase in V*_max_* of Cl^−^ absorption with no change in K*_m_*, indicating enhanced maximal transport capacity while substrate affinity remained unchanged. This kinetic pattern is characteristic of increased transporter abundance at the membrane rather than modification of intrinsic transport kinetics. Consistent with this interpretation, DRA and PAT1 mRNA levels were significantly elevated in intestinal villus cells from HFD-fed mice. This transcriptional upregulation was accompanied by increased total DRA/PAT1 protein expression and increased BBM localization, indicating that enhanced gene expression of DRA/PAT1 translates into greater protein synthesis and membrane insertion. Together, these data provide strong evidence that DIO stimulates DRA/PAT1-mediated chloride absorption primarily through transcriptional regulation.

Interestingly, in a genetic model of obesity, both DRA and PAT1 are downregulated in the distal colon [47]. In contrast, the current findings demonstrate upregulation of these transporters in DIO and specifically in the small intestine, suggesting that transporter regulation in obesity may be region-specific within the gastrointestinal tract. This region-specific difference suggests that distinct regulatory mechanisms may operate along different segments of the intestine, a possibility that should be explored further in our DIO model in future.

Functionally, increased DRA and PAT1 activity in DIO may enhance electroneutral chloride absorption and contribute to augmented NaCl absorption across the intestinal epithelium. Because DRA and PAT1 exchange luminal Cl^−^ for intracellular HCO_3_^−^, their increased activities may also influence luminal pH regulation and bicarbonate secretion. The increase in membrane-localized DRA and PAT1 proteins confirms that their transcriptional upregulation is accompanied by their functional incorporation into the apical membrane, thereby increasing their chloride absorptive capacity. However, the relative contribution of each of these transporters to elevated Cl^−^ absorption remains unresolved in the present study due to limitations in the methodology used to determine the Cl^−^ absorptive function. This limitation can be addressed in future studies using transporter-specific inhibitors or knockdown models that need to be performed to detangle how each contributes to the stimulated Cl^−^ absorption in obesity.

Our findings also demonstrate a significant decrease in basolateral Na^+^/K^+^-ATPase activity in villus cells from HFD-fed mice. Since Na^+^/K^+^-ATPase maintains the electrochemical Na^+^ gradient that drives SGLT1-mediated glucose uptake at the BBM, increased Na^+^ gradient generation cannot explain the observed increase in glucose transport. If anything, reduced Na^+^/K^+^-ATPase activity would be expected to diminish the driving force for sodium-coupled transport. Therefore, the enhanced glucose uptake observed in DIO must reflect intrinsic changes in SGLT1 functional efficiency at the apical membrane rather than enhanced transcellular Na^+^ gradient formation.

Despite the significant changes observed in SGLT1 and DRA/PAT1 activities, NHE3 activity and mRNA expression remained unchanged in DIO mice. This finding indicates that DIO does not uniformly stimulate all apical transport pathways within villus cells. Instead, intestinal transporter adaptation appears to involve selective stimulation of specific transport systems, each through distinct mechanisms, i.e., SGLT1 by altered *K_m_* and DRA/PAT1 by altered *V_max_*. This observation suggests that additional transport systems may be differentially regulated in DIO.

NHE3 plays a central role in electroneutral NaCl absorption by exchanging luminal Na^+^ for intracellular H, functionally coupled with Cl^−^/HCO_3_^−^ exchangers [48,49]. The absence of change in NHE3 activity suggests that the signaling pathways activated during DIO may preferentially target SGLT1 and DRA while sparing NHE3. Alternatively, preservation of NHE3 activity may represent a homeostatic mechanism to maintain intracellular pH and acid–base balance despite increased chloride absorption mediated by DRA/PAT1. Since NHE3 contributes to proton extrusion and intracellular pH regulation, maintaining stable NHE3 function may help preserve epithelial homeostasis under conditions of altered electrolyte transport. Additionally, dissociation between increased DRA/PAT1 activity and unchanged NHE3 function, as observed in this study and a previous study, suggests a partial uncoupling of the classical electroneutral NaCl absorption mechanism in obesity [50]. Such uncoupling may reflect differential regulation of transporter gene expression or post-translational signaling pathways within enterocytes.

Although the present study identifies transporter-specific changes underlying intestinal adaptation to DIO, several important limitations should be acknowledged. The observed alterations in transporter activity represent phenotypic consequences of HFD feeding, and the underlying molecular mechanisms were not directly examined. In particular, the pathways responsible for post-translational modulation of SGLT1 and transcriptional upregulation of DRA/PAT1 remain to be defined. Additionally, in vivo functional consequences of these changes, including their impact on whole-body glucose and electrolyte handling, were not assessed. Future studies using targeted mechanistic approaches and in vivo models will be required to further define the regulation and physiological significance of these transport adaptations in obesity.

## 4. Materials and Methods

### 4.1. Animal Models

Male C57BL/6 (B6) mice (4 weeks old) were obtained from The Jackson Laboratory (Bar Harbor, ME, USA) and used to establish a diet-induced obesity (DIO) model. Mice were randomly assigned to receive either a high-fat diet (HFD; 60% kcal from fat; TestDiet 58Y1, Cincinnati, OH, USA) ad libitum until 21 weeks of age or a low-fat diet (LFD; 10% kcal from fat; TestDiet 58Y2, Cincinnati, OH, USA) as controls. Animals were maintained under a 12-h light/dark cycle with free access to food and water. All procedures were approved by the Marshall University Institutional Animal Care and Use Committee (IACUC Protocol #732).

### 4.2. Villus Cell Isolation and Brush Border Membrane (BBM) Vesicle Preparation

Intestinal villus cells were isolated from the mid-jejunum and ileum either by Ca^2+^ chelation, as previously described, or by gentle mucosal scraping [51,52]. BBM vesicles (BBMV) were prepared using Mg^2+^ precipitation followed by differential centrifugation according to established protocols. Prepared BBMV were suspended in appropriate buffers for uptake assays or molecular analyses [52].

### 4.3. Sodium-Dependent Glucose Co-Transport Assays in Villus Cells and BBMV

SGLT1-mediated glucose uptake was assessed in intact villus cells and BBMV using the rapid-filtration technique with ^3^H-O-methyl-D-glucose (^3^H-OMG; generously provided by U. Sundaram, Marshall University Joan C. Edwards School of Medicine) as a non-metabolizable substrate [52]. For intact cell assays, 10 μL of villus cells were preincubated in Na^+^-free buffer (130 mM TMACl, 4.7 mM KCl, 1 mM MgSO_4_, 1.25 mM CaCl_2_, 20 mM Tris-HEPES, pH 7.4) and subsequently incubated in 90 μL reaction medium containing 130 mM NaCl, radiolabeled ^3^H-OMG (10 μCi), 100 μM OMG, and ±1 mM phlorizin. Uptake was terminated at 2 min by addition of ice-cold Na^+^-free stop solution containing 25 mM D-glucose. BBMV uptake assays were performed similarly, with uptake terminated at 60 s. Samples were filtered through Millipore HAWP filters (0.65 μm for intact cells; 0.45 μm for BBMV), washed, dissolved in scintillation fluid, and counted using a Beckman Coulter 6500 β-counter (Beckman, Brea, CA, USA). Kinetic parameters (V*_max_*, 1/K*_m_*) were derived from intact cell uptake assays performed with increasing OMG concentrations (0.5–50 mM) at 30 s using nonlinear regression (GraphPad Prism 8).

### 4.4. Na^+^/K^+^-ATPase Activity

Na^+^/K^+^-ATPase activity was quantified as inorganic phosphate (Pi) liberated from equal amounts of villus-cell homogenate under different experimental conditions, as previously described [53]. Enzyme activity was expressed as nmol Pi·mg^−1^ protein·min^−1^.

### 4.5. Cl^−^/HCO_3_^−^ and Na^+^/H^+^ Exchange Assays

BBMV were equilibrated in either vesicle medium 1 (VM1; 5 mM NMG gluconate, 50 mM HEPES-Tris pH 7.5, 100 mM KHCO_3_; gassed with 5% CO_2_/95% N_2_) or VM2 (5 mM NMG gluconate, 50 mM HEPES-Tris pH 7.5, 100 mM K-gluconate; gassed with 100% N_2_). Chloride uptake was initiated by adding 5 μL vesicles to 95 μL reaction buffer containing 5 mM NMG-^36^Cl^−^, 15 mM potassium gluconate, and 50 mM MES-Tris (pH 5.5), with or without 1 mM DIDS. Uptake was terminated at 1 min with ice-cold stop solution and quantified by scintillation counting. HCO_3_^−^-dependent, DIDS-sensitive ^36^Cl^−^ uptake was used to calculate Cl^−^/HCO_3_^−^ exchange activity. Kinetic analyses were performed in intact villus cells incubated with increasing HCl concentrations (0.5–50 mM) for 30 s.

Na^+^/H^+^ exchange activity was measured by incubating BBMV (5 μL) in reaction medium containing 300 mM mannitol, 50 mM Tris-HEPES (pH 7.5), and 1 mM ^22^NaCl, with or without 1 mM amiloride. Uptake was terminated at 1 min and quantified by rapid filtration and scintillation counting. ^22^Na and ^36^Cl were generously provided by U. Sundaram.

### 4.6. RT-qPCR

Total RNA was extracted from villus cells using the RNeasy Mini Kit (Qiagen, Germantown, MD, USA). cDNA was synthesized using the High-Capacity cDNA Reverse Transcription Kit (Applied Biosystems, Waltham, MA, USA). TaqMan^®^ assays (Thermo Fisher Scientific, Waltham, MA, USA)were used to quantify mouse *SLC5A1* (SGLT1) (Mm00451210_m1), *SLC26A3* (DRA) (Mm00445313_m1), *SLC26A6* (PAT1) (Mm00506742_m1), and *SLC9A3* (NHE3) (Mm01352473_m1), and β-actin (Mm01205647_g1), the latter serving as the endogenous control. Reactions were run for 40 cycles (95 °C for 15 s; 60 °C for 1 min). Assays were performed in triplicate using RNA from independent villus-cell preparations.

### 4.7. Protein Expression in Villus Whole Cell Lysates and BBM (Conventional Western Blotting and Capillary-Based Wes System)

Protein expression in villus whole-cell lysates and BBM was assessed using both conventional Western blotting and automated capillary immunoblotting (Wes, ProteinSimple, Bio-Techne brand, San Jose, CA, USA).

#### 4.7.1. Sample Preparation

Villus cells and BBMV were lysed in RIPA buffer supplemented with protease inhibitor cocktail (SAFC Biosciences, Lenexa, KS, USA). Protein concentrations were determined using the Bio-Rad DC^TM^ Protein Assay (Bio-Rad, Hercules, CA, USA) and verified using a NanoDrop 2000C spectrophotometer (Thermo Fisher Scientific, Waltham, MA, USA).

#### 4.7.2. Conventional Western Blotting

Equal amounts of protein were mixed with sample buffer, denatured, and separated on custom 8% SDS–polyacrylamide gels. Proteins were transferred to BioTrace PVDF membranes and blocked in 5% non-fat dry milk. Membranes were incubated with primary antibodies (1:1000 dilution) against SGLT1 (07-1417, Millipore Sigma, Burlington, MA, USA), DRA (sc-34939, Santa Cruz Biotechnology, Dallas, TX, USA), or PAT1 (sc-26728, Santa Cruz Biotechnology, Dallas, TX, USA), followed by HRP-conjugated secondary antibodies (mouse anti-rabbit sc-2357 or goat anti-mouse sc-2005 HRP, Santa Cruz Biotechnology, Dallas, TX, USA). Immunoreactive bands were visualized using enhanced chemiluminescence (ECL; Pierce, Rockford, IL, USA) and quantified by densitometry (FluorChem^TM^, Bio-Techne brand, San Jose, CA, USA).

#### 4.7.3. Capillary Immunoblotting (Wes System)

Capillary-based immunoblotting was performed using the Wes automated system (PN: SM-W004; ProteinSimple, Bio-Techne brand, San Jose, CA, USA) according to the manufacturer’s protocol. Protein lysates were mixed with Wes sample buffer, denatured, and loaded onto the assay plate along with primary antibodies (1:100 dilution) against SGLT1, DRA, and PAT1, the appropriate HRP-conjugated secondary antibodies, and the supplied chemiluminescent substrate. Protein separation, immunoprobing, and detection were carried out automatically within the capillary cartridges. Electropherogram peaks and virtual blot images were generated using Compass software Version 5.0.1(ProteinSimple, Bio-Techne brand, San Jose, CA, USA), and peak areas were quantified for analysis.

### 4.8. Plasma Glucose and Sodium Measurements

Plasma samples from LFD- and HFD-fed mice were submitted to the Clinical Laboratory Services at Cabell Huntington Hospital (Huntington, WV, USA) for measurement of sodium and glucose levels. The resulting data were used for statistical analysis.

### 4.9. Statistical Analysis

All uptake data are presented as mean ± SE. Each “*n*” represents triplicate uptake measurements performed using villus cells isolated from an individual mouse. Statistical significance between two groups was assessed using Student’s *t*-test, with *p* < 0.05 considered statistically significant.

## 5. Conclusions

Our study shows that DIO induces selective functional alterations of small intestinal transport processes through distinct molecular mechanisms. SGLT1 activity is enhanced via increased substrate affinity without changes in expression, whereas DRA/PAT1-mediated chloride exchange is stimulated through transcriptional upregulation and increased membrane abundance. NHE3 remains unaffected. These findings highlight transporter-specific adaptation in obesity and suggest that enhanced intestinal absorptive efficiency may contribute to metabolic dysregulation.

## Figures and Tables

**Table 1:** SGLT1 kinetic parameters in DIO mice villus cells.

Mice	V*_max_* (pmol/mg protein.30 s)	K*_m_* (mM)
**LFD**	3.04 ± 0.11	6.7 ± 0.80
**HFD**	3.02 ± 0.12	3.7 ± 0.58 *

The maximal velocity (V*_max_*) of glucose uptake of LFD and HFD-fed mice’s villus BBMV was unaffected. However, the affinity (1/K*_m_*) for glucose was significantly increased in HFD-fed mice villus BBMV compared to LFD-fed mice (*n* = 4; * *p* < 0.01).

**Table 2:** Cl^−^/HCO_3_^−^ exchange kinetic parameters in DIO mice villus cells.

Mice	V*_max_* (nmol/mg protein.30 s)	K*_m_* (mM)
LFD	0.97 ± 0.04	8.16 ± 0.9
HFD	1.24 ± 0.04 *	8.42 ± 0.8

The affinity (1/K*_m_*) for chloride uptake was unchanged between the diet groups. Conversely, the maximal velocity (V*_max_*) rate of chloride uptake was significantly stimulated in HFD-fed mice villus BBMV compared to LFD-fed mice (*n* = 3; * *p* < 0.01).

## Data Availability

All data supporting the findings of this study are included within the article.
